# Predictive Factors for the Efficacy of Radioactive Iodine Treatment of Graves' Disease: An Experience From 613 Chinese Patients

**DOI:** 10.1155/2024/7535093

**Published:** 2024-11-08

**Authors:** Wenwen Feng, He Shi, Yanli Yang, Jing Liu, Shiying Chen, Minghui Ren, Yajie Li, Wei Liu, Dai Cui

**Affiliations:** ^1^Department of Endocrinology, The First Affiliated Hospital of Nanjing Medical University, Nanjing, Jiangsu, China; ^2^Department of Geriatrics, The Affiliated Huaian Hospital of Xuzhou Medical University, Huaian, Jiangsu, China; ^3^Department of Nuclear Medicine, The First Affiliated Hospital of Nanjing Medical University, Nanjing, Jiangsu, China; ^4^Department of Geriatric Endocrinology, The First Affiliated Hospital of Nanjing Medical University, Nanjing, Jiangsu, China

## Abstract

**Objective:** The utilization of radioactive iodine-131I (RAI) has long been established as a cost-effective and conventional treatment for managing Graves' disease (GD). However, the accurate prediction of the clinical response to RAI treatment remains difficult. The successful resolution of GD through RAI therapy is typically characterized by the induction of hypothyroidism or euthyroidism. Thus, the principal aim of this study was to identify plausible predictors of RAI efficacy in the treatment of GD.

**Methods:** The clinical data of 613 GD patients, who underwent RAI treatment for the first time, were retrospectively analyzed, including age, gender, duration of hyperthyroidism, presence or absence of ocular signs, thyroid volume, thyroid weight, thyroid function (FT3, FT4, and TSH), radioactive iodine uptake (RAIU) at 2 h/6 h/24 h (2-h/6-h/24-h RAIU) prior to RAI treatment, the highest RAIU (RAIU_max_), and administered activity of 131I and 131I activity per gram of thyroid tissue. Success of RAI treatment was defined as achieving hypothyroidism or euthyroidism for more than 1 year after the initial treatment. Univariate and multivariate logistics regression analyses were conducted to identify factors that influence the efficacy of RAI treatment for GD. And at last, based on the results of the multivariate logistic regression analysis, a nomogram model was established.

**Results:** In this study, the success rate of RAI treatment for GD was 91.2% (559/613). Univariate analysis demonstrated that several factors, including age (*p*=0.005), thyroid volume (*p*=0.001), thyroid-stimulating hormone (TSH, *p*=0.042), ratio of RAIU at 6 h to 24 h (6-h/24-h RAIU, *p*=0.048), total 131I activity (*p*=0.026), and 131I activity per gram of thyroid tissue (*p*=0.001), were significantly associated with treatment outcome. Multivariate logistic regression analysis indicated thyroid volume and 131I activity per gram of thyroid tissue as significant independent predictors of radioactive iodine therapy (RIT) efficacy. The area under the ROC curve of the established nomogram model was 0.769 (95% confidence interval [CI]: 0.692–0.846), indicating that the model has good discriminatory ability.

**Conclusion:** Calculated-dose RAI is effective in the treatment of GD. The smaller thyroid volume and the higher 131I activity per gram of thyroid tissue are predictors of RAI efficacy in the treatment of GD.

## 1. Introduction

Hyperthyroidism is a pathological state characterized by the excessive synthesis and secretion of thyroid hormone (TH) by the thyroid gland, leading to heightened excitability and hypermetabolism in various physiological systems, such as the nervous, circulatory, and digestive systems. Prevalence studies estimate hyperthyroidism to affect 1.2%–1.6% of the population, with 0.5%–0.6% presenting with overt manifestations and 0.7%–1.0% being subclinical [1, 2]. The most frequent etiologies of this pathology include Graves' disease (GD), toxic multinodular goiter (TMNG), and toxic adenoma (TA) [2]. The current therapeutic options available for hyperthyroidism include antithyroid drugs (ATDs), radioactive iodine-131I (RAI) treatment, and thyroidectomy. Treatment modalities are selected based on factors such as geographical distribution, etiology, clinical status, and patient's preferences. In the United States, RAI is commonly the first-line therapy for GD, although there has been a recent trend towards increased preference for ATD. Meanwhile, in Europe, Latin America, and Japan, ATDs were more widely chosen for GD treatment [3]. RAI achieves its therapeutic effects through radiation-induced destruction of thyroid tissue. The ultimate goal of RAI treatment is to induce hypothyroidism or euthyroidism in patients. Despite its widespread use and high efficacy and safety, few well-designed prospective studies have evaluated its indications, optimal dose, efficacy, and side effects [4]. RAI is commonly indicated for patients who have failed to achieve remission or experienced recurrence with ATD therapy, suffered from serious ATD side effects, with high risk of surgery, or combined with hyperthyroid heart disease. In patients diagnosed with TMNG or TA, it is difficult to achieve remission with ATD alone, and RAI or surgery may be a more appropriate initial therapeutic option.

The success of RAI treatment for GD strongly depends on administered activities. It has been reported that 61% success was achieved with 5.4 mCi (200 MBq), 69% with 8.2 mCi (302 MBq), 74% with 10 mCi (370 MBq), 81% with 15 mCi (555 MBq), and 86% with 15.7 mCi (580 MBq) RAI in patients without adjunctive ATD [2]. However, RAI activities beyond a certain point does not improve the success rate of RAI therapy for severe Graves' hyperthyroidism [5] and may even increase the incidence of RAI therapy side effects. Clinically, the treatment activity of 131I can be achieved by either administering fixed activities or by calculating the activities based on the size of the thyroid and its ability to trap RAI. In addition, some studies have identified other factors affecting the success of RAI treatment for GD, such as age, gender, duration of hyperthyroidism, treatment with ATD, thyroid gland mass, serum-free thyroxine (FT4), thyrotropin receptor antibody (TRAb), radioactive iodine uptake (RAIU) at 24 h (24-h RAIU), 2-h RAIU, and ophthalmopathy at presentation [6–15].

In this study, we retrospectively evaluated the efficacy of calculated-dose RAI treatment in 613 patients with GD and analyzed factors associated with successful RAI treatment in the real world. We identified the independent risk factors affecting RAI treatment of GD through univariate analysis and multivariate logistic regression analysis for the clinical data of these 613 patients and established and verified a nomogram model through analysis software.

## 2. Materials and Methods

### 2.1. Patients

Totally, 613 patients with GD who were first treated with RAI at the Department of Nuclear Medicine, the First Affiliated Hospital of Nanjing Medical University, between 2011 and 2015, were retrospectively investigated. This study was approved by Ethics Committee of the First Affiliated Hospital of Nanjing Medical University (No. 2022-SR-278).

We collected the baseline clinical data of these patients before RAI treatment, including gender, age, duration of hyperthyroidism, presence or absence of ocular signs, thyroid volume, thyroid weight, thyroid function (FT3, FT4, and TSH), 2-h/6-h/24-h RAIU, the highest RAIU (RAIU_max_), ratio of RAIU at 2 h to 24 h (2-h/24-h RAIU), ratio of RAIU at 6 h to 24 h (6-h/24-h RAIU), total 131I activity (based on the calculation), and 131I activity per gram of thyroid tissue. All patients were interviewed by telephone in October 2021 (follow-up time varied from 70 to 130 months). The patient thyroid function status at 1 year after RAI treatment and at present were recorded, whether they were currently taking thyroid-related medications, had a recurrence of hyperthyroidism, or received a second RAI treatment. In addition, these patients were investigated for emerging diseases after RAI treatment, including tumor, immune-related diseases, hematological diseases, eye diseases, and adverse pregnancy outcomes (in women of reproductive age).

Prior to RAI treatment, all patients ceased ATDs for a period of 3–7 days. During this period, iodine diet should be avoided, such as avoiding iodized salt and iodine-rich foods. After RAI treatment, patients who were not contraindicated were prescribed beta-adrenergic blocking agents to prevent tachyarrhythmia. Success of RAI treatment was defined as achieving hypothyroidism or euthyroidism for more than 1 year after the initial treatment.

### 2.2. RAI Treatment Strategy

Before administering therapeutic RAI, the thyroid function test (FT3, FT4, and TSH), thyroid ultrasound, thyroid scintigraphy, and RAIU test are typically performed. The therapeutic 131I activity (*A*) is primarily determined by the thyroid weight (*G*), the highest RAIU (RAIUmax), and the quantity of radiation (*μ*Ci) to be deposited per gram of thyroid and calculated by the following formula: *A*=(*μ*Ci/*g* × *G*(*g*))/(RAIU_max_(%)).

Thyroid weight was evaluated by ^99m^TcO4^−^ thyroid scintigraphy, and RAIUmax referred to the highest iodine uptake rate within 24 h. The recommended quantity of 131I activity per gram of thyroid varies greatly, ranging from 50 to 200 *μ*Ci/g according to the American Thyroid Association (ATA) in 2016 [2]. In China, the commonly administered 131I activity per gram of thyroid tissue is 70–120 *μ*Ci/g (2.59–4.44 MBq) [16]. The formula assumes an effective half-life of 5 days, but adjustments may be made to the administered activity based on the patient's clinical condition or if the effective half-life differs significantly. In our center, the final administrated activity may be decreased for patients with higher thyroid weight or increased in order to eliminate the hyperthyroidism quickly.

### 2.3. Statistical Analysis

The statistical software used for data analysis and description was SPSS 26.0. The median and interquartile range were used for continuous data with abnormal distribution. The Mann–Whitney test was used to compare differences between two groups for nonnormally distributed data. Chi-square was used for categorical data analysis. Logistic regression analysis was carried out with variables that showed statistical significance for the outcome (*p* < 0.05). A *p* value of less than 0.05 was considered statistically significant, and all presented *p* values were two-tailed. The nomogram model, C-index, calibration curve, and ten-fold cross validation were obtained using R software package (4.1.3).

## 3. Results

### 3.1. Basic Clinical Characteristics of Patients before RAI Treatment

The clinical characteristics of all 613 patients (434 females and 179 males) are listed in Table 1. At the end of our follow-up, 91.2% (559/613) patients were hypothyroid or euthyroid after RAI treatment, who were defined as effective group. And 8.8% (54/613) patients were still suffered with hyperthyroid, defined as ineffective group.

### 3.2. Univariate Analysis of Influencing Outcomes of 131I Treatment for GD

By the Mann–Whitney test and chi-square test, we found that age (*p*=0.005), thyroid volume (*p*=0.001), thyroid-stimulating hormone (TSH, *p*=0.042), 6-h/24-h RAIU (*p*=0.048), total 131I activity (*p*=0.026), and 131I activity per gram of thyroid tissue (*p*=0.001) were significantly different between the two groups (Table 1). The results indicated that the patients who had successful treatment of GD were older, had smaller thyroid volume, lower TSH, and lower 6-h/24-h RAIU, and had been administered lower 131I activity and higher 131I activity per gram of thyroid tissue.

### 3.3. Multivariate Logistic Regression Analysis of the Factors on the Outcome of 131I Treatment for GD

Thyroid weight was removed from the regression model due to high variance inflation factor (VIF). After considering variables with *p* < 0.05 and performing collinearity diagnosis, multivariate logistic regression analysis showed that only thyroid volume and 131I activity per gram of thyroid tissue were significantly associated with the outcome of 131I treatment (step by step forward, listed in Table 2). The results indicate that the success rate of RAI treatment for GD is more likely to be higher with higher 131I activity per gram of thyroid tissue and smaller thyroid volume. Specifically, higher quantity of radiation deposited per gram of thyroid tissue and smaller thyroid volume can improve the efficacy of RAI treatment.

### 3.4. Establishment of the Nomogram Model for Predicting the Efficacy of RAI in the Treatment of GD

**Figure 1:**
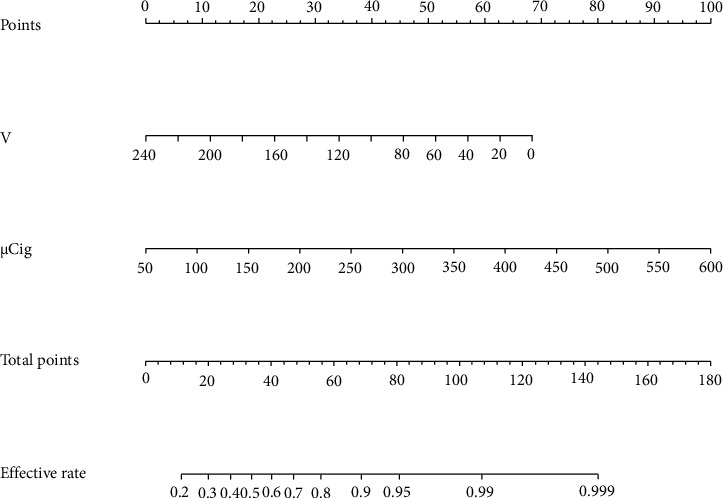
The nomogram model for predicting the efficacy of RAI in the treatment of Graves' disease. V: thyroid volume and *μ*Cig: 131I activity per gram of thyroid tissue.

**Figure 2:**
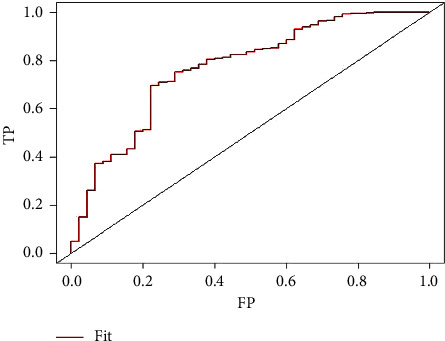
ROC curve verification of the nomogram model for predicting the efficacy of RAI in the treatment of Graves' disease. AUC: area under the curve, FP: false positive rate, and TP: true positive rate.

**Figure 3:**
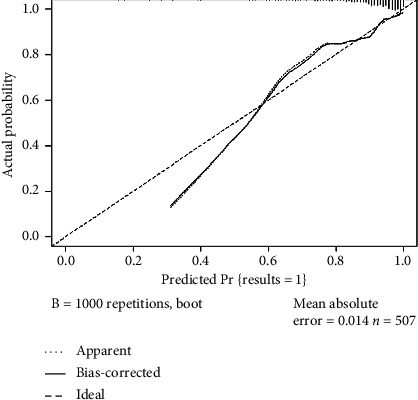
Calibration curve verification of the nomogram model for predicting the efficacy of radioactive iodine-131I in the treatment of Graves' disease.

A nomogram model was developed using the two factors identified from the multivariate logistics regression analysis to predict the efficacy of RAI in treating GD. The model was built using the R package and is shown in Figure 1. The area under the ROC curve was 0.769 with a 95% confidence interval of 0.692–0.846, indicating strong discrimination of the model as shown in Figure 2. The Hosmer–Lemeshow goodness-of-fit test had a chi-square value of 2.32 with a *p* value greater than 0.05, and the calibration curve is shown in Figure 3, confirming that the model has good prediction accuracy. In addition, we used the ten-fold cross-validation method to internally verify the regression model, and the results showed that the accuracy was 0.925 and the Kappa value was 0.234, indicating that the model had good stability.

### 3.5. Other Follow-Up Results

It is important to note that while RAI treatment is generally well tolerated, there can be potential risks and complications associated with the therapy. In our study, a total of 271 patients presented with ocular signs prior to RAI treatment, of which 15 (5.5%) experienced a worsening of ocular signs posttreatment. Furthermore, among the remaining 342 patients, 26 (7.6%) developed new ocular signs in the years following treatment. This indicates that a small proportion of patients (41/613, 6.7%) experienced exacerbations of ocular symptoms or the emergence of new ocular signs after receiving RAI treatment. In addition, we followed up on various potential adverse outcomes, including pregnancy complications, hematological disorders, tumor-related diseases, and immune-related disorders. Our findings indicate that a total of 78 women had 101 pregnancies, with 26 of these ended by induced abortions. Within the context of normal pregnancies, there were 62 (82.7%) successful full-term births and 13 (17.3%) adverse pregnancies, which included preterm delivery, spontaneous abortion, and intrauterine asphyxia. Leukopenia and thrombocytopenia were identified in 2 and 1 cases, respectively. Moreover, 4 cases were diagnosed with malignant tumors, and 4 patients were diagnosed with autoimmune diseases, specifically Sjogren's syndrome, myositis, and dermatomyositis.

## 4. Discussion

ATDs for GD have a remission rate of about 50% and require 1.5–2 years of treatment [17]; thus, many patients require RAI treatment. RAI therapy is typically reserved for patients who are intolerant to or do not respond well to ATDs, or have recurrent hyperthyroidism. Furthermore, RAI therapy remains a valuable treatment option for certain patients with GD due to its rapidity, simplicity, and cost-effectiveness. The target of RAI treatment is hypothyroidism, and the determination of radioiodine dose is very important. The method of dose determination includes standardized activity estimation and dosimetric calculation [18]. In our study, the administrated 131I activity was calculated by formula, and most of the final administrated 131I activity was adjusted empirically by clinicians based on dose calculation. A recent meta-analysis reported a success rate of approximately 79.8% for GD patients receiving 131I radiotherapy for the first time [6]. In our study, the response rate for Graves' hyperthyroidism treated with RAI for the first time was 91.2%, which is similar to the results of the Racaru LV's study (91% overall success rate for GD patients) [19].

The logistic regression analysis conducted in our study revealed that the success of RAI treatment is closely related to thyroid volume and the 131I administration activity per gram of thyroid. Thyroid volume is associated with radioactive iodine therapy (RIT) failure, which is also a risk factor for recurrence of GD treated with ATDs [1]. The larger the thyroid volume, the more destructive force it requires, and the dose of radioactive iodine administrated in practice tends to be conservative for various reasons, which is easy to lead to treatment failure. Many previous studies are consistent with our finding that the larger the thyroid volume, the greater the likelihood of RIT failure [19–21]. Shalaby et al. indicated that thyroid volume was positively associated with I-131 therapy failure in a meta-analysis [6]. The greater the 131I activity per gram of thyroid tissue, the greater the success rate of RIT. Similarly, Yu et al. reported that iodine dose per gram of thyroid tissue was an significant independent predictor of RIT efficacy [7] and higher iodine dose per gram of thyroid tissue was more likely to result in early hypothyroidism after radioiodine therapy in Yang et al.'s study [11]. This is easy to understand for the 131I activity per gram of thyroid eliminates the influence of thyroid weight, the greater the value, the stronger the destructive force, and the easier it is to develop hypothyroidism. Clinicians should fully understand these findings in clinical work, especially in the process of RIT. If the patient has a large thyroid volume, the administrated 131I activity can be adjusted according to the actual situation, and the appropriate 131I activity per gram of thyroid tissue can be selected.

Univariate analysis showed that multiple other factors were associated with treatment outcome. Age is an important factor affecting the efficacy of RAI treatment. Our study found that patients with successful treatment were generally older, which is consistent with Stachura et al.'s study [8]. This may be explained by younger patients' thyroid glands being more resistant to the destructive power of radioactive iodine. However, in another study, it was reported that higher therapeutic failure was observed among older patients [22]. Therefore, more studies are needed to confirm the effect of age on treatment outcomes. We also compared thyroid function in patients prior to RAI treatment and found that TSH was lower in patients who were successfully treated. We speculate that when TSH is low, the thyroid gland has a stronger iodine uptake function, and the destructive effect of radioactive iodine is stronger. Gland uptake is an important factor that affects the efficacy of RAI therapy, although its results are also controversial. Some studies have reported a higher risk of treatment failure with higher 24-h RAIU [6, 23], while others have shown a favorable outcome in patients with high 24-h RAIU [7, 14]. Our study did not find a statistically significant difference in the 24-h RAIU between the two groups, but it did find that the 6-h/24-h RAIU was lower in patients with successful treatment. The 6-h/24-h RAIU or 5-h/24-h RAIU can be used as a practical index for predicting rapid 131I turnover, and it is recognized as an important factor for predicting therapy outcome [24, 25]. In clinical practice, we often use the standard formula to calculate the 131I activity: obtained by thyroid weight and iodine uptake rate, the effective half-life of 131I will be ignored. The 5-h/24-h RAIU reflects the effective half-life of 131I to a certain extent. The higher the 5-h/24-h RAIU, the faster the 131I turnover rate. Rapid thyroidal 131I turnover will result in a shortened residence time of 131I in the thyroid gland and it can decrease the radiation dose delivered to the gland, which is a potential cause for therapeutic failure. Arora S reported that in high turnover GD patients, if administered standard 131I activity, the outcomes shall be poor. To improve the success rate, 131I activity should be increased by 1.5–2 times in the high turnover patients [26]. Similarly, other studies have also shown that lower 6-h/24-h RAIU was associated with early hypothyroidism [9] and high 5-h/24-h RAIU is positively associated with recurrence of hyperthyroidism following 131I therapy in Graves' hyperthyroidism [21]. We found that patients who were successfully treated for GD received lower activities of 131I. It seems that, owning to larger thyroid volume, the applied doses of 131I in unsuccessfully treated patients were still not high enough. This is consistent with the findings of a previous study by Šfiligoj et al. who reported that successful RAI treatment was associated with lower total 131I activity [20]. However, most previous studies have reported a positive correlation between the amount of applied 131I activity and the success rate of RAI treatment [27–29]. In short, there are more or less disputes about the influencing factors obtained by univariate analysis, which makes it difficult to predict the efficacy of RIT in the treatment of GD.

Theoretically, radiation therapy may have adverse effects on the human body, especially at high doses. However, current evidence does not suggest any adverse effects on long-term fertility, miscarriage, stillbirth, or birth defects in offspring [30, 31]. Our follow-up results showed an incidence of adverse pregnancy outcome at 17.3%. However, due to the lack of the control group and relevant clinical data of patients, horizontal comparison cannot be made. Guan L et al. found that radioiodine treatment of GD did not increase the risk of adverse pregnancy outcomes [32]. A systematic review was conducted by Acharya et al. compared RAI with ATD and concluded that RAI treatment was associated with an increased risk of developing or worsening ophthalmopathy compared to ATD [33]. In Acharya SH's study, the incidence of new or progressive ophthalmopathy was found to be 19.0% in RAI treatment for GD and 4.3% in ATD treatment. In our study, the incidence of ocular signs developing or worsening was 6.7%, which was not high compared to previous studies. This may be attributed to the subjective nature of our follow-up results, as patients were only asked about ocular symptoms by telephone, which could have led to recall bias due to the passage of time. The clinician cannot judge the actual condition of the eye sign, and the patient may provide wrong clinical data. It could also be related to the preventive use of glucocorticoids before RAI treatment. Without a control group, it is challenging to determine if the exacerbation of ocular symptoms or the emergence of new ocular signs is related to RAI treatment in our study. In the long run, RAI treatment did not have a significant impact on patients' quality of life.

One major limitation of this study is that the follow-up data are collected mainly from individual patient feedback rather than objective data, which makes the results less rigorous. Another limitation is the recall bias of patients interfering with the analysis of results, due to the time that has elapsed since RAI treatment (which occurred between 7 and 12 years ago). Finally, the study lacked some factors that needed to be included, such as thyroid function at initial diagnosis, whether or not accept ATDs treatment, treatment duration, and withdrawal time before RAI therapy.

## 5. Conclusion

In conclusion, calculated-dose RAI has been found to be an effective treatment for GD, with a high success rate closely linked to small thyroid volume and high 131I activity per gram of thyroid tissue. To further support and enhance these findings, additional studies with larger sample sizes and rigorous design are necessary.

## Figures and Tables

**Table 1:** Clinical characteristics of patients before RAI treatment: effective (hypothyroidism or euthyroidism) and ineffective (hyperthyroidism).

Variables	Hypothyroidism or euthyroidism	Hyperthyroidism	*p*
	(*n* = 559)	(*n* = 54)	
Sex (F/M)	395/164	39/15	0.810
Age (years)	42.00 (32.00, 49.00)	37.00 (26.50, 43.00)	0.005
Duration of hyperthyroidism (months)	36.00 (6.00, 84.00)	60.00 (12.00, 120.00)	0.079
Presence or absence of ocular signs (yes/no)	242/317	29/25	0.141
Thyroid volume (cm^3^)	49.00 (37.38, 68.13)	69.00 (52.00, 120.00)	0.001
FT3 (pmol/L)	14.60 (8.67, 22.50)	12.19 (6.73, 29.46)	0.381
FT4 (pmol/L)	39.20 (25.17, 56.82)	34.83 (17.37, 60.41)	0.238
TSH (mIU/L)	0.005 (0.005, 0.007)	0.005 (0.005, 0.021)	0.042
2-h RAIU (%)	46.49 (31.45, 61.36)	55.51 (33.71, 65.52)	0.141
6-h RAIU (%)	67.54 (53.62, 77.30)	73.35 (60.10, 80.20)	0.057
24-h RAIU (%)	71.26 (61.52, 79.20)	76.52 (61.56, 79.41)	0.240
RAIU_max_ (%)	73.00 (61.72, 80.30)	76.81 (60.33, 81.80)	0.292
2-h/24-h RAIU	0.62 (0.48, 0.79)	0.67 (0.45, 0.84)	0.444
6-h/24-h RAIU	0.93 (0.84, 0.99)	0.96 (0.86, 1.01)	0.048
Total 131I activity (mCi)	12 (8, 15)	13 (9, 15)	0.026
131I activity per gram of thyroid tissue (uCi/g)	191.08 (152.59, 241.94)	134.62 (117.55, 183.10)	0.001

Abbreviations: 2-h RAIU: 2-h radioactive iodine uptake; 6-h RAIU: 6-h radioactive iodine uptake; 24-h RAIU: 24-h radioactive iodine uptake; 2-h/24-h RAIU: ratio of radioactive iodine uptake at 2-h to 24-h; 6-h/24-h RAIU: ratio of radioactive iodine uptake at 6-h to 24-h; F: female; M: male; RAIU_max_: the maximum radioactive iodine uptake in 24 h.

**Table 2:** Multivariate logistics regression model: the efficacy of RAI in the treatment of Graves' disease.

Variables	*B*	Wald	*p*	OR	95% CI	
					Lower	Upper
Thyroid volume	−0.018	13.209	0.001	0.982	0.973	0.992
131I activity per gram of thyroid tissue	0.011	7.280	0.007	1.011	1.003	1.020
Constant	1.600	2.891	0.089	4.951		

## Data Availability

The data that support the findings of this study are available from the corresponding authors upon reasonable request.
